# Miscellaneous Intelligence

**Published:** 1847-08

**Authors:** 


ARTICLE XI.

### MISCELLANEOUS INTELLIGENCE.

We observe by an advertisement that the chair of Theory and Practice, vacated by Professor Moorehead in the Medical College of Ohio, has been filled by the transfer to it of Professor Harrison from the chair of Materia Medica, &c., and the appointment of our cotemporary of the Western Lancet, L. M. Lawson, M.D., to the latter.

Samuel H. Dickson, M.D., of Charleston, S. C., has been the successful applicant for the vacant chair of Theory and Practice in the University of the city of New York. He is distinguished as a writer on Pathology and Therapeutics.

Professor Henry of Princeton, New Jersey, has been appointed to the chair of Chemistry in the University of Pennsylvania, vacated by the resignation of Dr. Hare.

E. Deming, M.D., has been appointed to the chair of Materia Medica in Indiana Medical College.

M. L. Knapp, M.D., has been appointed to the chair of Materia Medica in St. Louis University, vacated by the resignation of Professor Norwood.

The first graduating class of the Medical Department of the University of Buffalo, at its recent commencement, numbered seventeen students.

The yellow fever is prevailing to an alarming extent in New Orleans. It is worse, and commenced earlier in the season, than for a number of years past.

The ship fever has been very fatal for several months amongst the half-famished immigrants that are landing in crowds at all of our principal seaport towns, and at Montreal and Quebec in Canada. It is thought by many to be contagious. Those attending upon the sick have many of them been infected and died of the disease.

Two cases of small pox occurred at Indianapolis in June and July, but it spread no farther.

A physician in Georgia has discovered a process by which cotton, and we suppose all ligneous fibre, may be rendered incombustible at a very moderate expense. It does not destroy the texture of the cotton. E.

